# Evaluation of Nutritional Status in Turkish Adolescents as Related to Gender and Socioeconomic Status

**DOI:** 10.4274/jcrpe.v2i3.111

**Published:** 2010-08-04

**Authors:** Işıl Özgüven, Betül Ersoy, Ali Aykan Özgüven, Pınar Dündar Erbay

**Affiliations:** 1 Celal Bayar University, School of Medicine, Department of Pediatrics, Manisa, Turkey; 2 Celal Bayar University, School of Medicine, Pediatric Endocrinology and Metabolism, Manisa, Turkey; 3 Celal Bayar University, School of Medicine, Department of Public Health, Manisa, Turkey; +90 236 232 31 33+90 532 626 77 92betul.ersoy@bayar.edu.trCelal Bayar University, School of Medicine, Pediatric Endocrinology and Metabolism, Manisa, Turkey

**Keywords:** adolescents, socioeconomic status, nutritional anthropometry, obesity

## Abstract

**Objective**: To evaluate the nutritional status of Turkish high school adolescents using anthropometric indicators and to determine the relationship of nutritional status with gender and socioeconomic status (SES) in adolescents.

**Methods**: Six hundred eighty adolescent students (n=284 males, 396 females) aged 14−18 years were selected from 6 high schools of different regions. Nutritional status was evaluated according to the anthropometric indicators, which were based on the WHO criteria. Adolescents were grouped into three SES categories.

**Results**: The rates of being stunted, underweight, and overweight/obesity were 4.4%, 5.0% and 16.8%, respectively. Height and weight standard deviation scores (SDS) were significantly lower in adolescents with low SES (p<0.05). The frequency of stunting was significantly higher in adolescents with low SES (p=0.012). Frequency of underweight, overweight and obesity did not differ significantly between socioeconomic groups and genders (p>0.05).

**Conclusion**: Adolescents of low SES were shorter and thinner than those of other SES categories. Undernutrition needs to be addressed in low SES. Among all Turkish adolescents, the major nutritional problems were overweight and obesity. There were no SES and gender differences in prevalence of overweight and obesity among the Turkish school adolescents living in urban areas. Prevalence of obesity is rising, regardless of differences in SES and gender, in developing countries too.

**Conflict of interest:**None declared.

## INTRODUCTION

Nutrition during adolescence plays an important role in the individual’s life. Malnutrition in adolescence encompasses undernutrition as well as overnutrition (1). Undernutrition implies being underweight for one's age, too short for one's age (stunted), or deficient in vitamins and minerals (2). Long−term undernutrition is an important cause of stunting or short height−for−age (3). Overnutrition implies being overweight for one's age and sex. Overweight/obesity is defined as excess body fat (2). There is strong evidence that childhood obesity is becoming increasingly prevalent in developing countries (4). The considerable amount of epidemiological data on child and adolescent obesity is in contrast to the paucity of data regarding the prevalence of thinness or underweight among adolescents (5).

Anthropometric measurements are often used to assess nutritional status in an individual or in communities (6). Limitations in establishing relations between anthropometric indicators and nutritional status in adolescents appear to have hindered research in this area (7). Despite the lack of a robust biological meaning and the difficulties in interpreting the cut−off values in adolescents, anthropometric indices are still the most common tools used in public health, particularly in developing countries (8). To our knowledge, there is a dearth of data on adolescent nutritional status in the urban areas of Turkey. The purpose of this study was to evaluate the nutritional status of Turkish high school adolescents using different anthropometric indicators. Nutritional status was also assessed with respect to gender and socioeconomic status (SES).

## METHODS

**Study Population and Design**

Design: Cross−sectional analysis using data from a prospective study. The study population consisted of 680 adolescent students (n=284 males, 396 females) aged 14−18 years, who were selected from high schools in different regions of the city of Manisa and represented individuals from a large range of SES categories and living conditions. The total number of students in Manisa high schools was 12688 during the 2005−2006 academic year. Permission was obtained from the Ministry of Education prior to the study. Five public high schools were selected randomly among fifteen public and four private high schools. The calculated sample size was 307, with 95% confidence interval, 3% deviation, and 8% prevalence. Obesity prevalence in Turkey was based on the calculation of sample size, but in our study the aim was to reach twice this sample size.

In this study, first of all, we identified the sociodemographic features of female and male adolescent students living Manisa. The adolescents were asked to describe the educational and occupational status of their parents, and their answers were confirmed by the school directors.

**Socioeconomic Status (SES)**

A questionnaire including a series of questions about their birthday, occupation and education of the parents was handed out to the adolescents. Categorization of socioeconomic class was based on the occupation and education of the parents by applying the Hollingshead index (Table 1) (8). Five educational levels and five occupational categories were used to identify socioeconomic classes. A score of 0 was given to the lowest and a score of 4 was given to the highest level of education and occupation. Three socioeconomic classes were identified, ranging from the lowest to the highest, on the basis of the sum of the scores. Hollingshead scoring was modified according to the national Turkish standards. The first and second socioeconomic classes in Hollingshead scoring were defined as low and middle, respectively, while the third and fourth classes were defined as high socioeconomic class.

**Anthropometric Measurements**

Weight was measured to the nearest 0.5 kg using a balance beam scale and height was measured to the nearest 0.1 cm with a manual height board. The body mass index (BMI; kg/m^2^) was used as an index of relative weight. Height standard deviation score (SDS), weight SDS, and BMI SDS were estimated according to the international standards (9). Adolescents suffering from chronic illnesses (thalassemia, type I diabetes mellitus, coeliac disease, etc.) were excluded from the study.

**Nutritional Status**

Classification of nutritional status was made according to the public health criteria recommended by a World Health Organization (WHO) expert committee (7). For analytic purposes, the subjects were divided into 4 groups: 1) normal, 2) low height−for−age (stunted), 3) low weightfor− age (underweight), 4) overweight and obese. A low height−for−age Z−score (HAZ or height SDS) (<−2SD) indicates stunting (stunted growth), and reflects a process of failure to reach linear growth potential as a result of suboptimal health and/or nutritional conditions. A low weight−for−age Z−score (WAZ, weight SDS) (<−2SD) is considered to indicate being underweight in the absence of significant wasting in a community (10, 11). More than 85th percentile of BMI for age and sex are used to define overweight and obesity (12).

**Statistical Analysis**

The mean values of anthropometric indices and indicators in the groups, classified according to SES, have been compared using an analysis of variance (one−way ANOVA). The group differences were evaluated by the Bonferroni post hoc test. The group differences in categorical variables were tested with χ^2^ test and χ^2^ for trend. Student’s t− and Mann−Whitney U tests were used for comparisons between two groups.

**Table 1:**
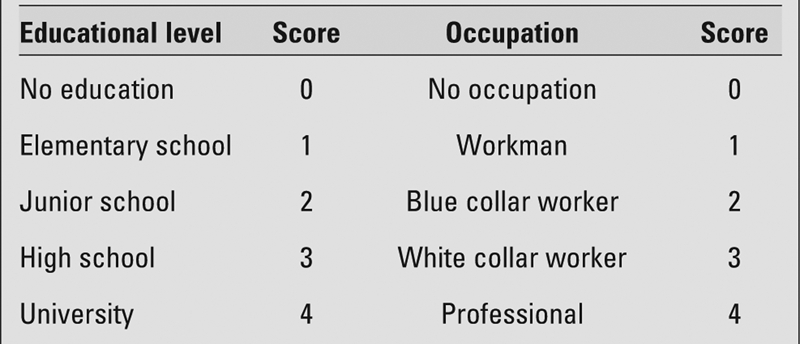
Scoring of the educational level and occupation of both parents according to the Hollingshead index for classification by socioeconomic groups

## RESULTS

In this study, an evaluation of the educational and occupational status of parents showed that while 14.1% of the fathers were university graduates, this rate was 6.6% for the mothers (Table 2). It was found that most of the mothers had only primary school education and they were housewives.

Table 3 shows the mean values for height SDS, weight SDS, BMI, and BMI SDS of adolescents according to their SES. All anthropometric indices showed significant differences according to SES. Height SDS values in adolescents of low SES groups were significantly lower compared to those in adolescents of high SES (p<0.001). BMI in adolescents of middle SES was found to be significantly higher than this in adolescents of other groups (p<0.001). However, BMI SDS did not change significantly according to SES (p=0.05). There was no significant difference in BMI SDS values between the groups according to SES (p>0.05).

**Figure 1:**
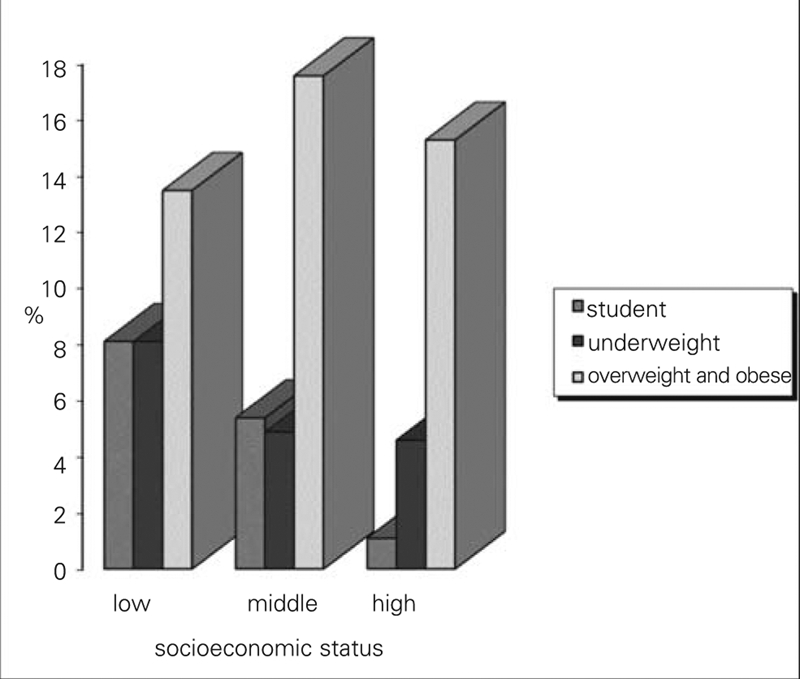
Frequency of being stunted, underweight, and overweight and obesity according to socioeconomic status (SES). The frequency of stunting was significantly higher in adolescents belonging low SES than those with high SES (p=0.012). The frequency of being underweight was not significantly different between socioeconomic groups (p=0.517). Frequency of being overweight and obese in adolescents belonging to middle SES was higher in adolescents belonging to other socioeconomic group, but it was not significant (p= 0.773).

Among all adolescents examined, 4.4% were found to be stunted and 5.0% to be underweight. However, 16.8% of all adolescents were overweight and obese. The frequency of stunting was significantly different between the socioeconomic groups (p=0.012). It was increased 4.96 times in adolescents of middle SES and 7.72 times in adolescents from low SES compared to those of high SES (χ^2^ trend=6.58, p=0.010) (Figure 1). Although frequency of being underweight in adolescents of low SES was higher than in adolescents of other SES groups, the difference was not significant (p=0.517). Frequencies of being underweight in adolescents of both high− and middle−SES categories were similar (Figure 1). Frequency of being overweight and obese in adolescents of middle SES was higher compared to that in adolescents belonging to other socioeconomic groups, but the difference was not significant (p= 0.773) (Figure 1).

**Figure 2:**
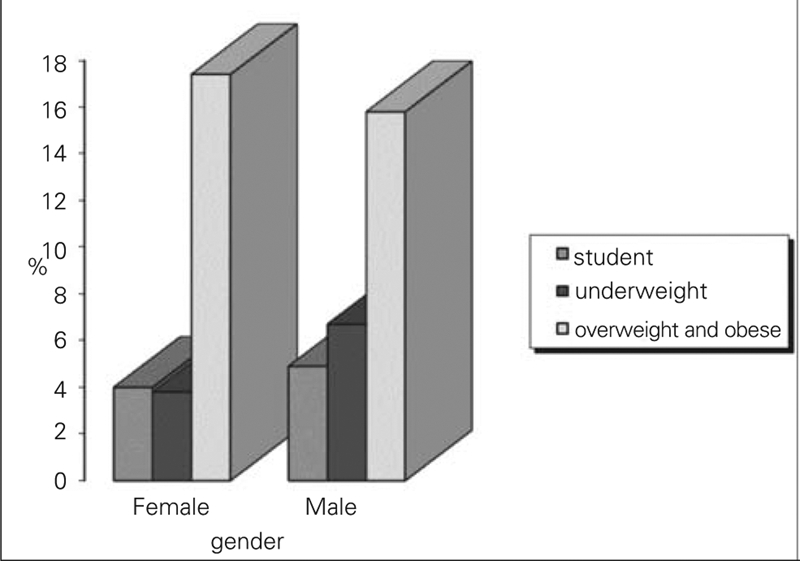
Frequency of being stunted, underweight, and overweight and obesity according to gender. Frequency of being stunted and underweight was not different significantly between male and female adolescents (p=0.354 and p=0.064 respectively). Frequency of being overweight and obese were higher in female adolescents, but there was no significant difference between two sexes (p=0.331).

We found that height and weight SDS values in male adolescents were not significantly different from those in female adolescents (p>0.05) (Table 4). Frequency of stunting was also not significantly different between male and female adolescents (p=0.354). Although frequency of being underweight in male adolescents tended to be higher than in female adolescents, the difference was not significant (p=0.063). We found that frequency of being overweight and obese tended to be higher in female adolescents, but no significant differences were observed between the two sexes (p=0.331) (Figure 2).

**Table 2:**
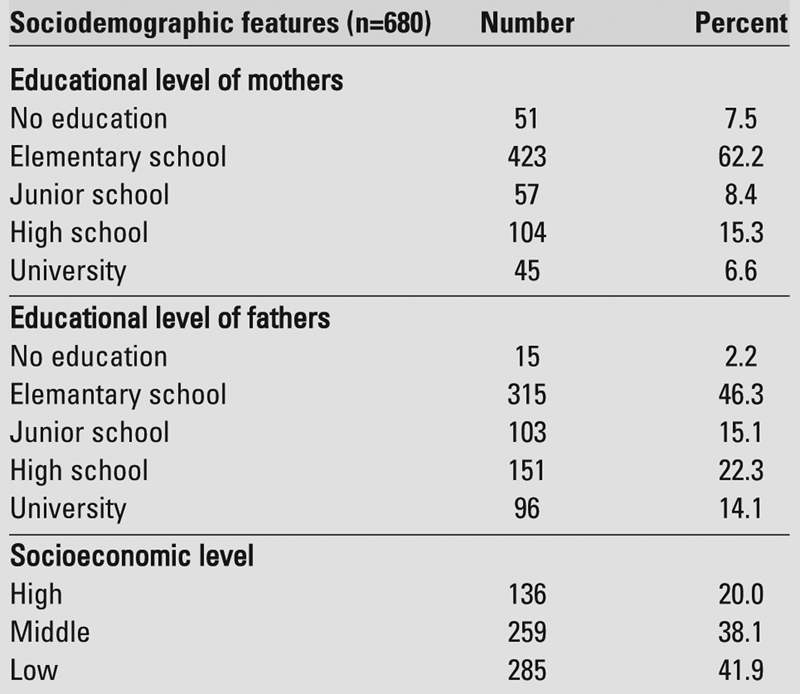
Distribution of adolescent girls and boys according to sociodemographic features

**Table 3:**
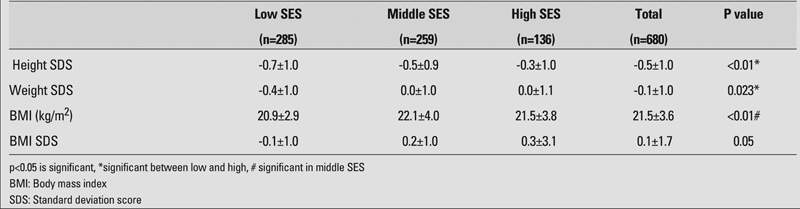
Anthropometric indices in adolescents according to socioeconomic status (SES)

**Table 4:**
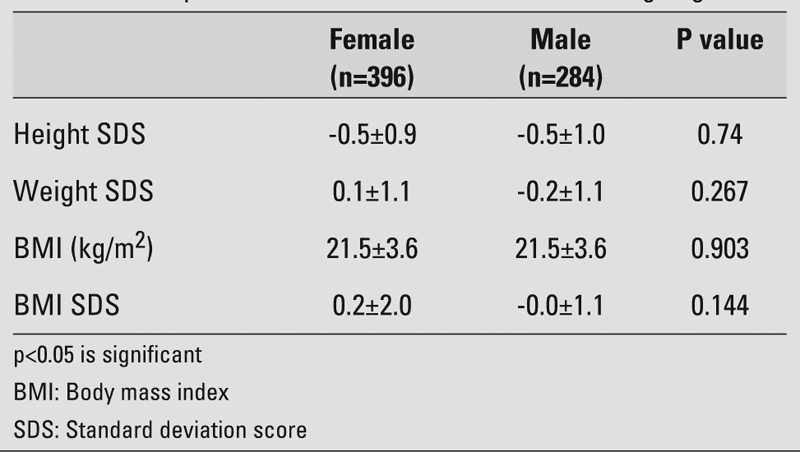
Anthropometric indices in adolescents according to gender

## DISCUSSION

Growth parameters, including height and weight, are highly heritable traits (13). Although genetic factors are significant, weight, fat mass, and fat distribution are influenced to a large extent by environmental factors (14). We compared anthropometric parameters of Turkish school adolescents belonging to different SES groups. Our study was performed in a city in the Aegean region in Western Turkey. Adolescents included in this study represented a general sample of the adolescent population of the city. In our country, more than 90% of adolescent boys and 80% of adolescent girls in urban areas attend high schools. Our results have shown that adolescents in low−SES groups were thinner and shorter than those in high−SES groups. Adolescents of middle SES were short, similar to adolescents in low−SES groups. However, weight in adolescents of middle SES was similar to that of adolescents of high SES. These results show that adolescents of middle SES are fatter than those in other SES groups.

Male adolescents were found to be taller and heavier than female adolescents. It is well known that among well−nourished children, there is a normal pattern of dimorphism where males tend to be taller and heavier than females. Growth references are different for males and females, thus, the observed sex difference might be related in some way to the reference itself. Because SDS values for height and weight are better markers in evaluation of height and weight, we assessed the anthropometric measurements of adolescents using SDS values. SDS values for anthropometric indices were not different in both male and female adolescents. In accordance with our results, the expected height SDS in adulthood was also not different between males and females, as also reported by others (15).

Much has been written about the current epidemic of child obesity (16), but undernutrition in children and adolescents poses considerably larger public health problem all over the world (17). Obesity and malnutrition represent pposite

extremes on the spectrum of adiposity, and both are routinely quantified in terms of weight and height relative to the child's age (18). In this study, we evaluated the nutritional status of adolescents at high school based on the WHO criteria. Among all adolescents, frequency of being stunted and underweight were 4.4% and 5%, respectively. In a previous study, we found that stunting (7.5%) was more prevalent than underweight (4.1%) in children of primary school age (19). In a study from Africa, stunting and wasting are reported as the most commonly encountered signs of nutritional deficiency, unlike the results of the present study (20). In our study, stunting was more prevalent in adolescents belonging to low−SES groups. This result suggests that chronic undernutrition, as indicated by deficit in height, decreased with increasing income level. Unlike our study, the rate of underweight was significantly higher at the low−income level among 2 to 18 years old males in Pakistan in a study reported by Hakeem (21).

We found that overweight and obesity were the most prevalent nutritional disorders in our study group. The frequency of overweight/obesity was 16.8% among all adolescents. Adolescence has been identified as a critical period for the development of overweight and obesity and there is strong evidence that this state persists into adulthood (22). There has been a strong interest in studying the relationships between SES and obesity. In general, the literature shows that in industrialized countries, low−SES groups are more likely to be obese than their high−SES counterparts, whereas high−SES groups are at increased risk for obesity in developing countries (23, 24, 25). We found that prevalence of overweight and obesity in Turkish adolescents were not significantly different between the socioeconomic groups. The changing weight trends shown by the present study poses a major challenge for lower−income countries. Current nutrition programs for adolescents should be reviewed and revised to consider these rapidly emerging concerns. A study from Africa shows that the prevalence of overweight and obesity was higher in females, particularly in post−menarcheal girls (26). In contrast with this result, both higher prevalence and increment of overweight and obesity among boys in urban areas was reported in a study from China (27). In our study, there were no sex differences in the prevalence of overweight and obesity among adolescents.

In conclusion, our study shows that height and weight status improve with a higher SES in adolescents of both sexes. Adolescents of low SES were shorter and thinner than those in other SES groups. Anthropometric measurements in adolescents of middle SES were similar to those of adolescents in high−SES groups. This result indicates that the differences between middle− and high−SES groups in height and weight status are decreasing. However, nutrition−related disorders and particularly undernutrition need to be addressed in low−SES groups. Among the Turkish adolescents, the major nutritional problems are overweight and obesity. Prevalence of obesity is high and rising in the Western regions of Turkey. Higher prevalence of overweight and obesity in Turkish adolescents may be due to increasing urbanization and changes in the structure of the diet. Prevalence of obesity is rising, regardless of differences in SES and gender also in other developing countries. Further longitudinal studies are needed to fully understand the underlying causes of overweight and obesity in adolescence and to be able to and to take preventive measures.
